# Supplementary material for: A web-database of mammalian morphology and a reanalysis of placental phylogeny
Source: BMC Evol Biol. 2007 Jul 3;7:108. doi: 10.1186/1471-2148-7-108 (PMC1941728; doi:10.1186/1471-2148-7-108)
Supplement: Additional File 1 — Website data: The 196 characters first described in [17], along with the supplementary data indicated below, have been joined into a number of HTML files, all of which are linked to the "morphsite_bmc07.html" index page. The embedded links to the supporting files are functional, independent of PC or platform, provided that the directory structure remains intact (i.e., all files remain in a single folder, no subdirectories). Nexus files: Datafiles containing the morphological, indel, and aligned DNA sequence data, linked from the "morphsite_bmc07.html" index page. Table 1. Changes to the multiple sequence alignment of [9]. Table 2. Qualitative character-state corrections to the morphology matrix of [17]. Table 3. Summary of discrepancies between Appendices 1 and 2 of [17]. [file 1471-2148-7-108-S1.zip › asher-BMC07-website/web-litcited.html]

Adkins,
R.M., Walton, A.H., Honeycutt, R.L. (2003). Higher-level systematics
of rodents and divergence time estimates based on two congruent
nuclear genes. *Mol. Phyl. Evol.* **26:** 409-420.

Agur
and Lee, (1991). *Grant's Atlas of Anatomy*, 9th Edition.
Williams and Wilkins, Baltimore.

Amrine,
H. M. and Springer, M. S. (1999). Maximum likelihood analysis of the
tethythere hypothesis. *J. Mamm. Evol.* **6:** 161-176.

Andrews,
C. W. (1906). A descriptive catalogue of the Tertiary Vertebrata of
the Fayum, Egypt. British Museum Natural History: London.

Arnason,
U., Addegoke, J.A., Bodin, K., Born, E.W., Esa, Y.B., Gullberg, A.,
Nilsson, M., Short, R., Xu, X., Janke, A. (2002). Mammalian
mitogenomic relationships and the root of the eutherian tree. *Proc.
Natl. Acad. Sci. USA* **99:** 8151-8156.

Asher,
R. J. (1999). A morphological basis for assessing the phylogeny of
the "Tenrecoidea" (Mammalia, Lipotyphla). *Cladistics*
**15:** 231--252.

Asher,
R.J. (2000). *Phylogenetic History of Tenrecs and Other
Insectivoran Mammals*. Ph.D. Dissertation, State University of
New York, Stony Brook.

Asher,
R.J. (2001). Cranial anatomy in tenrecid insectivorans: character
evolution across competing phylogenies. *American Museum Novitates*
**3352:** 1-54.

Asher,
R. J. (2003). Phylogenetics of the Tenrecidae (Mammalia): a response
to Douady et al. 2002. *Mol. Phyl. Evol.* **26:**
328-330.

Barnes,
L. G. (1990). The Fossil Record and Evolutionary Relationships of the
Genus *Tursiops*. In: *The Bottlenosed Dolphin*, S.
Leatherwood and R.R. Reeves, eds., pp. 3-26, Academic Press, New
York.

Beard,
K. C. and MacPhee, R. D. E. (1994). Cranial anatomy of *Shoshonius*
and the antiquity of the Anthropoidea. In: *Anthropoid Origins*,
Fleagle, J. G. and Kay, R. F., eds., pp. 55-97, Plenum, New York.

Butler,
P. M. (1956). The skull of *Ictops* and the classification of
the Insectivora. *Proc. Zool. Soc.London* **126:** 453-481.

Butler,
P. M. (1988). Phylogeny of the insectivores. in *Phylogeny
and Classification of the Tetrapods Vol. 2* ed.
M. J. Benton (Clarendon Press, Oxford), pp. 117--141.

Carter,
A. M. (2001). Evolution of the Placenta and fetal membranes seen in
the light of molecular phylogenetics. *Placenta*, **22:**
800-807.

Carter
AM, Enders AC. (2004).  Comparative aspects of
trophoblast development and placentation. Reprod Biol Endocrinol.
2:46.

Cartmill,
M. (1978). The orbital mosaic in prosimians and the use of variable
traits in systematics. *Folia Primatologica* **30**(2):
89--114.

Cartmill,
M. (1980). Morphology, function, and evolution of the anthropoid
postorbital septum. In: *Evolutionary Biology of the New World
Monkeys and Continental Drift*. Ciochon, R.L. and Chiarelli, A.B.,
eds., pp. 243-274, Plenum, New York.

Cartmill,
M. and R. D. E. MacPhee. (1980). Tupaiid affinities: the evidence of
the carotid arteries and cranial skeleton. In: *Comparative
Biology and Evolutionary Relationships of Tree Shrews*. P.
Luckett, ed., pp. 95-132, Plenum, New York.

Cifelli,
R. (1982). The petrosal structure of *Hyopsodus* with respect
to that of some other ungulates, and its phylogenetic implications.  *J. Paleontology* **56:** 796-805.

Clark,
J. M. (1991) A new early Miocene species of *Paleoparadoxia*
(Mammalia: Desmostylia) from California. *J. Vertebr.
Paleontol.* **11:** 490-508.

Colless,
D. H. (1980) Congruence between morphometric and allozyme data for
*Menidia* species: a reappraisal. *Syst. Zool.* **29**(3):
288-299.

Court,
N. (1990). Periotic anatomy of *Arsinoitherium* (Mammalia,
Embrithopoda) and its phylogenetic implications. *J. Vertebr.
Paleontol.* **10:** 170-182.

Court,
N. (1992). The skull of *Arsinoitherium* (Mammalia,
Embrithopoda) and the higher order phylogenetic relationships of
ungulate mammals. *Palaeovertebrata (Montpelier)*. **22:**
1-43*.*

Court,
N. (1993). Morphology and functional anatomy of the postcranial
skeleton in *Arsinoitherium* (Mammalia, Embrithopoda).
*Palaeontographica (Stuttgart) A.* **226:** 125-169.

Court,
N. (1994). The periotic of *Moeritherium* (Mammalia,
Proboscidea): homology or homoplasy in the ear region of Tethytheria
McKenna, 1975. *Zool. J. Linn. Soc.* **112:** 13-28.

Court,
N. (1995). A new species of *Numidotherium* (Mammalia,
Proboscidea) from the Eocene of Libya and the early phylogeny of the
Proboscidea. *J. Vertebr. Paleontol.* **15:** 650-671.

Dathe,
F. (1982). *Megaptera hubachi* n. sp., ein fossiler Bartenwahl
aus marinen Sandsteinschichten des tieferen Pliozäns Chiles. *Z.
Geol. Wiss.* **11:** 813-848.

Domning,
D., Ray, C. E., M. C. McKenna. (1986). Two new OIigocene
desmostylians and a discussion of tethytherian systematics.
*Smithson. Contrib. Paleobio.* **59:** 1-56.

Douady,
C.J., Catzeflis, F., Kao, D.J., Springer, M.S., and Stanhope, M.J.
(2002a) Molecular Evidence for the Monophyly of Tenrecidae (Mammalia)
and the Timing of the Colonization of Madagascar by Malagasy Tenrecs.
*Mol. Phyl. Evol.* , **22:**
357-363.

Douady,
C.J., Chatelier, P.I., Madsen, O., de Jong, W.W., Catzeflis, F.,
Springer M.S., Stanhope, M.J. (2002b) Molecular phylogenetic
evidence confirming the Eulipotyphla concept and in support of
hedgehogs as the sister group to shrews. *Mol. Phyl. Evol.* **25:**
200-209.

Douady,
C.J., Catzeflis, F., Springer, M.S., and Stanhope, M.J. (2003)
Phylogenetics of the Tenrecidae (Mammalia): a reply to Asher. *Mol.
Phyl. Evol.* **26:**
331-332.

Downton,
M. and Austin, A.D. (2002) Increased congruence does not necessarily
indicate increased phylogenetic accuracy. *Syst. Biol.* **51:**
19-31.

Fischer,
M. S. (1986). Die Stellung der Schliefer (Hyracoidea) im
phylogenetischen System der Eutheria. *Cour. Forsch. Inst.
Senckenberg.* **84:** 1-132.

Fischer,
M. S., 1989: Hyracoids, the sister-group of perissodactyls. 37-56. *in*
Prothero, D. R. & Schoch, R. M., (eds.) 1989: The Evolution of
Perissodactyls. Oxford University Press, Oxford.

Fischer,
M. S. (1998). Die Lokomotion von *Procavia capensis* (Mammalia:
Hyracoidea): zur Evolution des Bewegungssystems bei Saugetieren.
*Abhandlungen des Naturwissenschaftlichen Vereins in Hamburg.*
**33:** 1-188.

Fischer,
M. and P. Tassy. (1993). The interrelation between Proboscidea,
Sirenia, Hyracoidea, and Mesaxonia: the morphological evidence. In:
*Mammal Phylogeny*, volume 2: Placentals. F. S. Szalay, M. J.
Novacek and McKenna, M. C. Eds., pp. 217-234, Springer-Verlag, New
York.

Fleagle,
J. G. (1999). *Primate Adaptation and Evolution*, 2nd edition.
Academic Press, San Diego.

Felsenstein,
J. (1978) Cases in which parsimony or compatibility methods will be
positively misleading. *Syst. Zool.* **27:** 401-410.

Felsenstein,
J. (1988) Phylogenies from molecular sequences: inference and
reliability. *Ann. Rev. Genet.* **22:**
521-565.

Frost,
D. R., Rodrigues, M. T., Grant, T., and Titus, T. (2001)
Phylogenetics
of the lizard genus *Tropidurus* (Squamata: Tropiduridae). 
*Mol. Phylogenet. Evol.* **21**, 352--371.

Frost,
D. R., Wozencraft, C., and Hoffman, R. S. (1991). Phylogenetic
relationships of hedgehogs and gymnures (Mammalia: Insectivora:
Erinaceidae). *Smithson. Contrib. Zool.* 518, 1-69.

Gatesy,
J., R. DeSalle and W. Wheeler. (1993) Alignment ambiguous nucleotide
sites and the exclusion of systematic data. *Mol. Phyl. Evol.*
**2,** 152-157.

Gatesy,
J., Mathee, C., DeSalle, R., Hayashi, C. (2002) Resolution of a
Supertree/Supermatrix Paradox. *Syst. Biol.* **51**(4),
652--664.

Gaudin,
T.J., Wible, J.R., Hopson, J.A., Turnbull, W.D. (1996) Reexamination
of the morphological evidence for the cohort Epitheria (Mammlia,
Eutheria) *J. Mamm. Evol*. **3,** 31--79.

Gazin,
C. L. (1956) A study of the Eocene condylarthran mammal *Hyopsodus. Smithson. Misc. Collect.* **131**(6):
1--57.

Gazin,
C. L. (1965). A study of the early Tertiary condylarthran mammal
*Meniscotherium*. *Smithson. Misc. Collect.* **149:**
1--98.

Gazin,
C. L. (1968). A study of the Eocene condylarthran mammal *Hyopsodus. Smithson. Misc. Collect.* **153:** 1--90.

Geisler,
J. H. (2001). New morphological evidence for the phylogeny of
Artiodactyla, Cetacea, and Mesonychidae.
*Am. Mus. Novit.* 3344: 1--53.

Geisler, J.H., and
Luo, Z. (1996). The petrosal and inner ear of *Herpetocetus* sp.
(Mammalia: Cetacea) and their implications for the phylogeny and
hearing of archaic mysticetes. Journal of Paleontology 70(6):
1045-1066.

Geisler,
J. H. and Luo, Z. (1998). Relationships
of Cetacea to terrestrial ungulates and the evolution of ranial
vasculature in Cete. In: *The Emergence of Whales,* J. G. M.
Thewissen, Ed., pp. 163-212, Plenum, New York.

Gheerbrandt,
E., Sudre, J., Iarochene, M., Moumni, A. (2001). First ascertained
African "condylarth" mammals from the earliest Ypresian of
the Ouled Abdoun Basin, Morocco. *J. Vertebr. Paleontol.* **21:**
107-118.

Giribet,
G. Edgecomb, G. D., and Wheeler, W.C. (2001). Arthropod phylogeny
based on eight molecular loci and morphology. *Nature* **413:**
157-161.

Goloboff,
P. A. (1999). NONA computer program version 2.0. Fundación e
Instituto Miguel Lillo, Tucumán, Argentina. Download at
www.cladistics.org.

Gregory,
W. K. 1910. The orders of mammals. *Bulletin American Museum of
Natural History* **27:** 1--524.

Grzimek
(1975). *Grzimek's Animal Life Encyclopedia*, I-IV. Van
Nostrand Rheinhold: New York.

Hildebrand,
M. 1985. Digging of quadrupeds, pp. 89-109. *In* M. Hildebrand,
D. M. Bramble, K. F. Liem, and D. B. Wake (eds.), Functional
Vertebrate Morphology. Belknap, Cambridge.

Hermanson,
J. W. and B. J. MacFadden (1996). Evolutionary and functional
morphology of the knee in fossil and extant horses (Equidae). *J.
Vertebr. Paleontol.* **16:** 349-357.

Horovitz,
I. (1999) A phylogenetic study of living and fossil platyrrhines
*Am. Mus. Novit.* **3269:**
1-40.

Horovitz,
I. (2000) The tarsus of *Ukhaatherium nessovi* (Eutheria,
Mammalia) from the Late Cretaceous of Mongolia: an appraisal of the
evolution of the ankle in basal therians. *J. Vertebr. Paleontol.*
**20:** 547--560.

Ijiri,
S. and Kamei, T. 1961. [in Japanese] On the skulls of *Desmostylus
mirabilis* Nagao from South Sakhalin and of *Palaeoparadoxia
tabatai* Tokunaga from Gifu prefecture, Japan *Earth Science*
53, 1-27.

Janies,
D. A. and Wheeler, W.C. (2001) Efficiency of parallel direct
optimization. *Cladistics* **17:**
S71-S82.

Johnson,
P. A. and Fox, R. C. (1984). Paleocene and Late Cretaceous mammals
from Saskatchewan, Canada. *Palaeontographica Abt. A* **186:**
163--222.

Kamei,
T., Okazaki, Y. (1975). Neogene desmostylid and proboscidean fossils
from Japan. *Atlas of Japanese Fossils* **34**(199 and 201):
1-4.

Keller,
J. (1990). *Wale und Delphine* (Jahr Verlag, Hamburg).

Kielan-Jaworowska,
Z. (1979). Evolution of the therian mammals in the Late Cretaceous
of Asia. Part III. Postcranial skeleton in Zalambdalestidae.
*Palaeontol. Pol.*, **38:** 3-41.

Kielan
Jaworowska, Z. (1984). Evolution of the therian mammals in the Late
Cretaceous of Asia. Part V. Skull structure in Zalambdalestidae
*Palaeontol. Pol.* **46:**
107-117.

Kindahl,
M. (1959). Some aspects of the tooth development in
Soricidae. Acta Odontologica Scandanavica 17: 203–237.

Krause,
D. W., R. R. Rodgers, C. A. Forster, J. H. Hartman, G. A. Buckley
and S. D. Sampson. (1999). Late Cretaceous vertebrate fauna of
Madagascar: implications for Gondwanan paleobiogeography. *GSA
Today* **9:** 1-7.

Lee,
M. S. Y. (2001) Unalignable sequences and molecular evolution.
*Trends Ecol. Evol.* **16:**
681--685.

Lin,
Y-H., Waddell, P., Penny, M. (2002a) Pika and vole mitochondrial
genomes increase support for both rodent monophyly and glires. *Gene*
**294:** 119-129.

Lin,
Y-H, McLeanachan, P.A., Gore, A.R., Phillips, M.J., Ota, R., Hendy,
M.D., Penny, M. (2002b) Four new mitochondrial genomes and the
increased stability of evolutionary trees of mammals from improved
taxon sampling. *Mol. Biol. Evol.* **19:** 2060-2070.

Lillegraven,
J. A. (1969). Latest Cretaceous mammals of upper part of Edmonton
Formation of Alberta, Canada, and review of marsupial-placental
dichotomy in mammalian evolution. *Univ. Kansas Paleontol.
Contrib.* **50** (vertebrata 12): 1-122.

Lillegraven,
J. A. (1984). Why was there a marsupial-placental dichotomy? In:
*Mammals: Notes from a Short Course*. Gingerich, P. D. and
Badgley, C. E., Eds., pp. 72-86, Univ. Tenessee Dept. Geol. Sci.
Studies in Geology, vol. 8, Knoxville.

Lillegraven,
J. A. (1985). Use of the term "trophoblast" for tissues in
therian mammals. *J. Morphol.* **183:** 293-299.

Lillegraven, J. A.
2004. Polarities in mammalian evolution seen through homologs of the
inner cell mass. J. Mammal. Evol. 11:143–202.

Luckett,
W.P. 1977. Ontogeny of amniote fetal membrances and their application
to phylogeny. In: *Major Patterns in Vertebrate Evolution*. M.K.
Hecht, P.C. Goody, and B.M. Hecht, Eds., pp. 439-516, Plenum, New
York.

Luckett,
W.P. (1985). Superordinal and intraordinal affinities of rodents:
developmental evidence from the dentition and placentation. In:
*Evolutionary Relationships among Rodents*. W.P. Luckett and
J-L. Hartenberger, Eds., pp. 227-276, Plenum Press, New York.

Luckett,
W.P. (1993). Uses and limitations of mammalian fetal membrances and
placenta for phylogenetic reconstruction. *J. Exp. Zool.* **266:**
514--527.

Luo,
Z. (1998). Homology and transformation of cetacean ectotympanic
structures. In: *The Emergence of Whales*, J.G.M. Thewissen,
ed., pp. 269-301, New York, Plenum.

Luo,
Z., and Gingerich, P. D. (1999). Terrestrial Mesonychia to aquatic
Cetacea: transformation of the basicranium and evolution of hearing
in whales. *Univ. Mich. Pap. Paleontol.* **31:** 1-98.

MacPhee,
R. D. E. (1979.) Entotympanics, ontogeny, and primates. *Folia
Primatol.* **31:** 23-47.

MacPhee,
R. D. E. (1994). Morphology, adaptations, and relationships of
*Plesiorycteropus*, and a diagnosis of a new order of eutherian
mammals. *Bull. Am. Mus. Nat. Hist.* **220:** 1--214.

MacPhee,
R. D. E. and M. J. Novacek. (1993). Definition and relationships of
Lipotyphla. In *Mammal Phylogeny, volume 2: Placentals.* F. S.
Szalay, M. J. Novacek and M. C. McKenna, Eds., pp. 13--31, Springer
Verlag, New York.

MacPhee,
R. D. E., Novacek, M. J. and Storch, G. (1988). Basicranial
morphology of Early Tertiary erinaceomorphs and the origin of
primates. *American Museum Novitates* **2921:**
1--42.

Madsen,
O., Scally, M., Douady, C. J., Kao, D. J., DeBry, R. W., Adkins, R.,
Amrine, H. M., Stanhope, M. J., de Jong, W. W., and Springer, M. S.
(2001). Parallel adaptive radiations in two major clades of placental
mammals. *Nature* **409:** 610--614

Mahboubi,
M., Ameur, J. Y, Crochet, Y., and Jaeger, J. J. (1986). El Kohol
(Saharan Atlas, Algeria), a new Eocene mammal locality in
northwestern Africa. *Palaeontographica Abt. A* **192:**
15--49.

Malia,
M. J., Adkins, R. M., and Allard, M. W.
(2002). Molecular support for Afrotheria and the polyphyly of
Lipotyphla based on analyses of the growth hormone receptor gene.
*Molec. Phylogenet. Evol.* **24:** 91--101.

Matsumoto,
H. (1923). A contribution to the knowledge of *Moeritherium*.
*Bull. Am. Mus. Nat. Hist.* **48:** 97--139.

McDowell,
S. B., Jr. (1958). The Greater Antillean insectivores. *Bull. Am.
Mus. Nat. Hist.* **115:** 113--214.

McKenna,
M. C. (1963). New evidence against tupaioid affinities of the
mammlian family Anagalidae. *Am. Mus. Novit.* **2158:**
1--16.

McKenna,
M. C. and Bell S. K. (1997). Classification of Mammals Above the
Species Level (Columbia Univ. Press, New York).

McKenna,
M. C. and Manning, R. (1977). Affinities and palaeobiogeographic
significance of the Mongolian Paleogene genus *Phenacolophus*.
*Geobios Mem. Spec.* **1:** 61-85.

Meng,
J. and Wyss, A. (2001). The morphology of *Tribosphenomys*
(Rodentiaformes, Mammalia): phylogenetic implications for basal
Glires. *J. Mamm. Evol.* **8:** 1--72.

Meng,
J. Hu, Y., and Li, C. (2003) The osteology of *Rhombomylus*
(Mammalia: Glires): implications for the phylogeny and evolution of
Glires. *Bull. Am. Mus. Nat. Hist.* **275:** 1--247.

Mickoleit,
G. (2004). *Phylogenetische Systematik der Wirbeltie**re*
(Pfeil Verlag, Munich).

Millar,
J. S. (1981). Pre-partum reproductive characteristics of eutherian
mammals. *Evolution*. **35:** 1149--1163.

Mossman,
H. W. (1987). *Vertebrate Fetal Membranes.* Rutgers Univ.
Press, New Brunswick.

Mouchaty,
S.K, Gullberg, A., Janke, A., and Arnason, U. (2000). Phylogenetic
position of the tenrecs (Mammalia: Tenrecidae) of Madagascar based on
analysis of the complete mitochondrial genome sequence of Echinops
telfairi. *Zoologica Scripta* **29**:
307-317.

Murphy,
W. J., Elzirik, E., Johnson, W. E., Zhang, Y. P., Ryder, O. A., and
O'Brien, S. J. (2001a) Molecular phylogenetics and the origin of
placental mammals. *Nature* **409:** 614-618.

Murphy,
W. J., Eizirik, E., O'Brien, S.J., Madsen, O., Scally, M., Douady,
C.J., Teeling, E., Ryder, O.A., Stanhope, M.J., deJong, W.W.,
Springer, M. S. (2001b). Resolution of the early placental mammal
radiation using Baysian phylogenetics. *Science* **294:**
2348--2351.

Novacek,
M. J. (1977). Aspects of the problem of variation, origin, and
evolution of the eutherian auditory bulla. *Mammal. Rev.* **7:**
131--149.

Novacek,
M. J. (1986) The skull of leptictid insectivorans and the
higher-level classification of eutherian mammals. *Bull. Am. Mus.
Nat. Hist.* **183:**
1--111.

Novacek,
M. J. (1989). Higher mammal phylogeny: the morphological-molecular
synthesis. In *The Hierarchy of Life,*
Fernholm, B., Bremer, K., and Jörnvall, H. (eds.), pp.
421--435, Elsevier, Amsterdam.

Novacek,
M. J. (1991). "All tree histograms" and the evaluation of
cladistic evidence: some ambiguities. *Cladistics* **7:**
345--349.

Novacek,
M. J. (1992). Mammal phylogeny: shaking the tree. *Nature* **356:**
121--125.

Novacek,
M. J., Rougier, G.W., Wible, J.R., McKenna, M.C., Dashzeveg, D.,
Horovitz, I. (1997).
Epipubic bones in eutherian mammals from the late Cretaceous of
Mongolia. *Nature* **389:** 483--486.

Novacek,
M. J. & Wyss, A. R. (1986). Higher level relationships of the
recent eutherian orders: the morphological evidence. *Cladistics*
**2:** 257--287.

Novacek,
M. J., Wyss, A, and McKenna, M. C. (1988). The major groups of
eutherian mammals. In: *Phylogeny and Classification of Tetrapods*.
M. J. Benton (Ed.), pp. 31--71, Clarendon, Oxford.

O'Leary,
M. O. and J. H. Geisler. (1999). The position of Cetaceous within
Mammlia: phylogenetic analysis of morphological data from extinct and
extant taxa. *Syst. Biol.* **48:** 455--490.

Pol,
D., and Siddall, M.E. (2001). Biases in maximum likelihood and
parsimony: A simulation approach to a 10-taxon case. *Cladistics*
**17**(3): 266-281.

Radinsky,
L. B. (1969). The early evolution of the Perissodactyla. *Evolution*
**23:** 308-328.

Reinhart,
R. H. (1959). A review of the Sirenia and Desmostylia. *Univ.
Calif. Pub. Geol. Sci.* **36:** 1--146.

Renfree,
M. B. (1993). Ontogeny, fenetic control, and phylogeny of female
reproduction in monotreme and therian mammals. In: *Mammal
Phylogeny, Vol. 1,* F. S. Szalay, M. J. Novacek, M. C. McKenna,
Eds., pp. 4--20, Springer, New York.

Rose,
K. D. and Emry, R. J. (1993). Relationships of Xenarthra, Pholidota
and fossil "edentates": the morphological evidence. In
*Mammal Phylogeny, Vol. 2,* F. S. Szalay, M. J. Novacek, M. C.
McKenna, Eds., pp. 81--102, Springer, New York.

Ross,
C. F. (1995). Muscular and osseous anatomy of the primate anterior
temporal fossa and the functions of the postorbital septum. *Am.
J. Phys. Anthro.* **98:** 275--306.

Ross,
C. F., Williams, B., and Kay, R. F., 1998. Phylogenetic analysis of
anthropoid relationships. *J. Hum. Ev.* **35:** 221-306.

Schulmeister,
S., Wheeler, W. C., and Carpenter, J. M. (2002). Simultaneous
analysis of the basal lineages of Hymenoptera (Insecta) using
sensitivity analysis. *Cladistics* **18:** 455--484.

Schutt,
W. A. and Simmons, N. B. 1998. Morphology and homology of the
chiropteran calcar, with comments on the phylogenetic relationships
of *Archaeopteropus*. *J. Mamm. Evol.* 5, 1-32.

Shikama,
T. 1966. Postcranial skeletons of Japanese demostylians. *Paleontol.
Soc. Japan Sp. Paper* 12, 1-202.

Shoshani,
J. and McKenna, M. C. 1998. Higher taxonomic relatiobships among
extant mammals based on morphology, with selected comparisons of
results from molecular data. *Mol. Phylo. Evol.* 9, 572-584.

Shoshani,
J., West, R.M., Court, N., Savage, R.J.G., Harris, J.M. (1996) The
earliest proboscideans: general plan, taxonomy, and palaeoecology.
In: *The Proboscidea: Evolution and Paleoecology of Elephants and
Their Relatives* Shoshani, J. and Tassy, P. eds., pp. 57--75.
(Oxford, Oxford University Press).

Siddall,
M.E. (1998). Success of parsimony in the four-taxon case: long-branch
repulsion by likelihood in the Farris zone. *Cladistics* **14**(3):
209-220.

Siddall,
M.E. and Whiting, M..F. (1999). Long branch abstractions.
*Cladistics* **15:** 9--24.

Simmons,
N. B. 1994. The case for chiropteran monophyly. *Am. Mus.
Novitates*, **3103:** 1-54.

Simmons,
N. B. 1995. Bat reltionships and the origin of flight. *Symposia
of the Zoological Society of London* **67:** 27-43.

Simmons,
N. B. and J. H. Geisler. 1998. Phylogenetic relationships of
*Icaronycteris*, *Archaeonycteris*, *Hassianycteris*,
and *Palaeochiropteryx* to extant bat lineages, with comments of
the evolution of echolocation and foraging strategies in
Microchiroptera. *Bull. Am. Mus. Nat. Hist.* **235:**
1--182.

Simons.
E.L. and Rasmussen, D. T. (1989). Cranial morphology of
*Aegyptopithecus* and *Tarsius* and the question of the
tarsier-anthropoidean clade. *Am. J. Phys. Anthro*., **79:**
1--23.

Simpson,
G. G. (1931). A new insectivore from the Oligocene, Ulan Gochu
horizon of Mongolia. *Am. Mus. Novitates* **505:** 1.

Simpson,
G. G. (1945). The principles of classification and a classification
of mammals. *Bull. Am. Mus. Nat. Hist.* **85:** 1--350.

Smith,
A.B. (1998). What does paleontology contribute to systematics in a
molecular world? *Mol. Phyl. Evol.* **9**(3):
437--447.

Springer,
M. S., Murphy, W.J., Eizirik, E., O'Brien, S.J. (2003). Placental
mammal diversification and the Cretaceous Tertiary boundary. *Proc.
Natl. Acad. Sci. USA* **100:** 1056--1061.

Stanhope,
M. J., Waddell, V.G., Madsen, O., deJong, W.W., Hedges, S.B., Cleven,
G.C., Kao, D., Springer, M.S. (1998). Molecular evidence for
multiple origins of the Insectivora and for a new order of endemic
African mammals. *Proc. Natl. Acad. Sci. USA* **95:**
9967--9972.

Starck,
D. (1995). *Lehrbuch der Speziellen Zoologie; Band II: Wirbeltiere;
Teile 1-2, Saeugetiere*. Gustav Fischer Verlag, Jena.

Swofford, D. L.
(2000). Phylogenetic Analysis Using Parsimony (\*and Other Methods)
Computer Program Version 4.0b10. Sinauer Associates, Sunderland,
Massachusetts.

Swofford,
D.L., Waddell, P.J., Huelsenbeck, J.P., Foster, P.G., Lewis, P.O.,
Rogers, J.S. (2001). Bias in Phylogenetic Estimation and Its
Relevance to the Choice between Parsimony and Likelihood Methods.
*Syst. Biol.* **50**(4):525--539.

Szalay,
F. S. and Lucas, S. (1993). Cranioskeletal morphology of archontans,
and diagnoses of Chiroptera, Volitantia, and Archonta. In: *Primates
and Their Relatives in Phylogenetic Perspective,* R. D. E.
MacPhee, Ed., pp. 187--226. New York: Plenum.

Tabuce,
R., Coiffait, B., Coiffait, P-E., Mahboubi, M., Jaeger, J-J. (2001).
A new genus of Macroscelidea (Mammalia) from the Eocene of Algeria: a
possible origin of elephant shrews. *J. Vertebr. Paleontol.* **21:**
535--546.

Tassy,
P. (1981). Le crane de *Moeritherium* (Proboscidea, Mammalia)
de l'Eocene de Dor el Talha (Libye) et le probleme de la
classification phylogenetique du genre dans les Tethytheria. *Bull.
Mus Natn. Hist. nat*. **3:** 87--147.

Thewissen,
J. G. M. (1989). Mammlian frontal diploic vein and the human foramen
caecum. *Anat. Rec*. **223:** 242--244.

Thewissen,
J. G. M. (1990). Evolution of Paleocene and Eocene Phenacodontidae
(Mammalia, Condylarthra). *Univ. Mich. Pap. Paleontol.* **29:**
1--107.

Thewissen,
J. G. M. (1994). Phylogenetic aspects of cetacean origins: a
morphological perspective.  *J. Mammal. Evol.* **2:**
157--184.

Thewissen,
J. G. M., & Domning, D. (1992). The role of phenacodontids in the
origin of the modern orders of ungulate mammals. *J. Vertebr.
Paleontol*. **12:** 494.

Thompson,
J. D., T. J. Gibson, F. Plewniak, F. Jeanmougin and D. G. Higgins.
(1997). The CLUSTAL-X windows interface: Flexible strategies for
multiple sequence alignment aided by quality analysis tools. *Nucleic
Acids Research* **25:** 4876-4882.

True,
F. W. (1904). The whalebone whales of the western North Atlantic.
*Smithson. Contrib. Knowledge* **33:** 1--332.

Tyndale-Biscoe C.H. 2004. Australasian marsupials - to cherish and to
hold. Reproduction Fertility and Development 13 (7-8): 477-485.

Vanderhoof,
V. L. (1937). A study of the Miocene sirenian *Desmostylus*.
*Univ. Calif. Pubs. Geol. Sci.* **24:** 119--262.

van
Dijk, M.A.M, Paradis, E., Catzeflis, F., and deJong, W.W. (1999).
The virtues of gaps: xenarthran (edentate) monophyly supported by a
unique deletion in A-crystallin. *Syst. Biol.*
**48:** 94--106.

Webb,
S. D. and Taylor, B. E. (1980). The phylogeny of hornless ruminants
and a description of the cranium of *Archaeomeryx*. *Bull.
Am. Mus. Nat. Hist.* **167:**121--157.

Werdelin,
L. and Nilsonne, A. (1999). The evolution of th scrotum and
testicular descent in mammals: a phylogenetic view.  *J. Theor.
Biol.* **196:** 61--72.

Westheide,
W., Rieger, R.M. (2003). *Spezielle Zoologie, Band 2, Wirbeltiere*
(Spektrum Akademischer Verlag)

Wheeler,
W. C. (1995). Sequence alignment, parameter sensitivity, and the
phylogenetic analysis of molecular data. *Syst. Biol*. **44:**
321--331.

Wheeler,
W. C. (1996). Optimization alignment: the end of multiple sequence
alignment in phylogenetics? *Cladistics* **12:** 1--9.

Wheeler,
W. C. (1999). Measuring topological congruence by extending character
techniques *Cladistics*
**15:** 131--136.

Wheeler,
W. C. (2001). Homology and the optimization of DNA sequence data.
C*ladistics* **17:**
S3--S11.

Wheeler, W.C.,
Gladstein, D., and DeLaet, J. (2002) POY direct optimzation computer
program, versions test\_5 through 3.0.5. Available at
ftp.amnh.org/pub/molecular/poy.

Wible,
J. R. and Novacek, M. J. 1988. Cranial evidence for the monophyletic
origin of bats. *Am. Mus. Novitates* **2911:** 1--19.

Wible,
J. R., Novacek, M. J., and Rougier, G. W. (2004). New Data on the
Skull and Dentition in the Mongolian Late Cretaceous Eutherian Mammal
*Zalambdalestes.* *Bull. Am. Mus. Nat. Hist.* **281**:1--144.

Wible
J. R. and Rougier, G. W. 2000. Cranial anatomy of *Kryptobataar
dashzevegi* (Mammlia, Multituberculata) and its bearing on the
evolution of mammalian characters. *Bull. Am. Mus. Nat. Hist.*
**247:** 1--124.

Wible
J. R., Rougier, G. W., Novacek, M. J., and McKenna, M. C. 2001.
Earliest eutherian ear region: a petrosal referred to *Prokennalestes*
from the Early Cretaceous of Mongolia. *Am. Mus. Novitates*
**3322:** 1--44.

Williamson,
T. E. and Lucas, S. G. (1992). *Meniscotherium* (Mammalia,
"Condylarthra") from the Paleocene-Eocene of western North
America. *New Mex. Mus. Nat. Hist.* *Bull.* **1:**
1--75.

Wislocki,
G.B. (1930).
On an Unusual Placental Form in the Hyracoidea:
Its Bearing on the
Theory of the Phylogeny of the Placenta. Contributions to Embryology
21 (122):83-95.

Wyss,
A. D. (1987). Notes on Proteutheria, Insectivora, and Thomas
Huxley's contribution to mammalian systematics. *J. Mamm.* **68:** 135--138

Wyss,
A. R. and J. J. Flynn. (1993). A phylogenetic analysis and definition
of the Carnivora. In: *Mammal Phylogeny, volume 2:
Placentals*, F. S. Szalay, M. J.
Novacek and M. C. McKenna, Eds., pp. 32--52, Springer-Verlag, New
York.

Yalden,
D. W. 1966. The anatomy of mole locomotion. J. Zool., Lond.
149:55-64.
